# Palaeoproteomic and genetic insights into millennial-scale dairy consumption in Armenia

**DOI:** 10.1016/j.isci.2026.117829

**Published:** 2026-10-09

**Authors:** Mariya Antonosyan, Anahit Hovhannisyan, Patrick Roberts, Lara M. Cassidy, Satenik Mkrtchyan, Hasmik Simonyan, Arsen Bobokhyan, Levon Aghikyan, Ashot Piliposyan, Ruben Badalyan, Nzhdeh Yeranyan, Artak Gnuni, Hayk Avetisyan, Mikayel Badalyan, Armine Gabrielyan, Narineh Sarkisian, Avetis Grigoryan, Hakob Simonyan, Meri Safaryan, Benik Vardanyan, Kristine Martirosyan-Olshansky, Iseult Jackson, Emily Breslin, Andranik Gyonjyan, Zaruhi Khachatryan, Jana Ilgner, Levon Yepiskoposyan, Andrea Manica, Daniel G. Bradley, Nicole Boivin, Shevan Wilkin

**Affiliations:** 1 Department of Coevolution of Land Use and Urbanisation, Max Planck Institute of Geoanthropology, 07745 Jena, Thuringia, Germany; 2 Smurfit Institute of Genetics, Trinity College Dublin, D02 VF25 Dublin 2, Ireland; 3 Institute of Molecular Biology, NAS Armenia, Yerevan 0014, Armenia; 4 Institute of Archaeology and Ethnography, National Academy of Sciences, Yerevan 0025, Armenia; 5 Service for the Protection of Historical Environment and Historical Cultural Museum Reservations, Ministry of Education, Science, Culture and Sports, Yerevan, Armenia; 6 Chair of Archaeology and Ethnography, Yerevan State University, Yerevan 0025, Armenia; 7 Institute of History, National Academy of Sciences, Yerevan 0019, Armenia; 8 Scientific Research Centre of the Historical and Cultural Heritage, Ministry of Education, Science, Culture and Sports, Yerevan 0010, Armenia; 9 Cotsen Institute of Archaeology, University of California, Los Angeles, Los Angeles, CA 90095, USA; 10 Scientific Center of Zoology and Hydroecology, National Academy of Sciences, Yerevan 0014, Armenia; 11 Laboratory Facility, Max Planck Institute of Geoanthropology, 07745 Jena, Thuringia, Germany; 12 Department of Zoology, University of Cambridge, Cambridge CB2 3EJ, UK; 13 Max Planck Institute of Geoanthropology, 07745 Jena, Thuringia, Germany; 14 School of Social Science, University of Queensland, Brisbane, QLD 4072, Australia; 15 Griffith Sciences, Griffith University, Nathan, QLD 4111, Australia; 16 Department of Environmental Sciences, University of Basel, 4056 Basel, Basel-Stadt, Switzerland; 17 Institute of Evolutionary Medicine, University of Zurich, 8057 Zürich, Switzerland; 18 Australian Research Centre for Human Evolution, Griffith University, Brisbane, QLD 4111, Australia

**Keywords:** dairying, lactase persistence, Armenia, South Caucasus

## Abstract

Dairy products are a key component of diet in Armenia today, yet the origins of milk consumption, its historical development, and genetic adaptations related to milk digestion remain understudied. Here, we investigate the co-evolution of dairying practices and lactase persistence in Armenia through combined palaeoproteomic and genetic analyses spanning prehistory to the present. Our palaeoproteomic results provide direct evidence of milk consumption in prehistoric Armenia and, with existing regional data, indicate that dairying was an established component of the South Caucasus subsistence economy at least from the 4th millennium BCE. Milk proteins were predominantly recovered from high-altitude sites, suggesting that dairy consumption may have served as an adaptive strategy in highland environments. In contrast, genetic findings indicate that lactase persistence-associated variants never reached high frequencies throughout Armenian prehistory. This may reflect cultural adaptation to fermenting dairy products, reducing lactose content and enabling dairy consumption in genetically lactose-intolerant populations.

## Highlights

• Integrated palaeoproteomic and genetic study of dairying in Armenia over 6,000 years
• Milk consumption in prehistoric Armenia since at least the 4th millennium BCE
• Multi-species dairy use; dairy proteins predominantly recovered from high-altitude sites
• Cultural practices enabled sustained dairying without widespread genetic adaptation

## Graphical abstract

**Figure:**
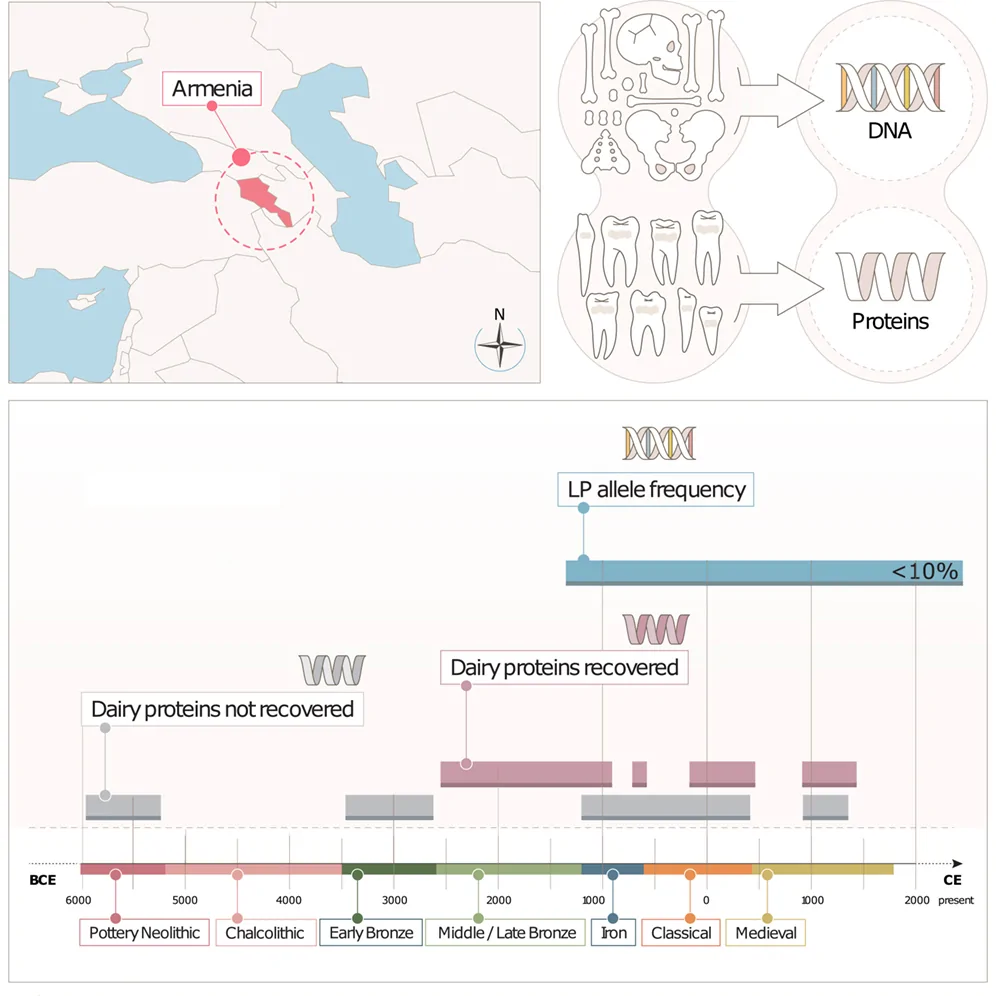

## Teaser

Anthropology; archeology; biological sciences; genomics; omics; proteomics

## Introduction

The incorporation of animal milk into human diets was a major transition in prehistoric societies, enabling the supply of nutrients without slaughtering precious livestock. In marginal environments, milk additionally often functioned as a source of hydration and nutrients, supporting adaptive strategies in resource-scarce settings.^1^^,^^2^ Moreover, the processing of milk into dairy products allowed for the production of food sources that could be stored and transported over long periods, sustaining mobile pastoral groups.^3^ These functional advantages of dairy products have been suggested to further boost the long-term resilience and demographic success of groups that integrated dairy into their diets.^4^^,^^5^^,^^6^ Postweaning access to abundant sources of milk would not have been widely available to humans without the domestication of such milk-producing species as cattle, sheep, and goats. Zooarchaeological evidence, particularly that based on herd management and culling patterns, suggests that dairying may have emerged as early as the 8th millennium BCE,^7^^,^^8^ shortly after the emergence of the first domesticated animals in the Near East.^9^ Meanwhile, direct evidence for dairy consumption in the form of milk lipids present as organic residues in archaeological pottery revealed that ruminants were exploited for their milk from at least the 7th millennium BCE onwards in Anatolia,^10^^,^^11^^,^^12^ with this practice subsequently spreading across other regions.^1^^,^^3^^,^^13^^,^^14^^,^^15^^,^^16^^,^^17^

Located at the crossroads linking Mesopotamia and the Near East to the broader Eurasian continent, the South Caucasus region, including Armenia, served as a vital corridor for the exchange of people, knowledge, and subsistence practices, including the spread of milk consumption northwards to the Pontic steppe by the 5th millennium BCE.^18^ This early tradition of milk use and processing is reflected in the enduring importance of dairy in the region. Today, dairy products are an essential part of daily diets in Armenia, with both fresh milk and processed dairy consumed regularly. Traditional processing follows three primary methods: fermentation (to transform fresh milk into yoghurts, cheeses, sour creams, curd, kerif, koumiss, etc.), additive coagulation (to produce cheeses), or cream separation (to produce sour cream).^19^ Lactic acid fermentation is particularly widespread and central to local food culture, exemplified by *matsun*, Armenia’s traditional yoghurt.^20^ In addition to being consumed as a fermented food, *matsun* also plays a versatile role in Armenian cuisine, serving as the base for *t’an*, a refreshing summer drink, and as a key ingredient in *spas*, a traditional soup made with grains and yoghurt. When dried on bread (*čòrat’an*), *matsun* can also be used as a starter culture for baking and when churned, it yields butter. Despite this rich dairy heritage, remarkably little is known about the origin and historical development of dairying practices in the region, a gap that palaeoproteomic analysis is uniquely positioned to address.

Adult milk consumption and lactose digestion are made possible by postweaning production of the enzyme lactase-phlorizin hydrolase, which hydrolyses the milk sugar lactose into its component monosaccharides. Not all human populations possess the lactase persistence (LP) phenotype required to digest and absorb lactose into adulthood. Its presence has been hypothesized to be linked to the consumption of prehistoric milk, reflecting co-evolution between cultural innovations and genetic control of lactase production.^21^^,^^22^ LP is associated with at least five independently evolved SNPs—13910C>T (rs4988235), −13907C>G (rs41525747), −13915T>G (rs41380347), −14009T>G (rs869051967), and −14010 G>C (rs145946881)—located in the *MCM6* gene’s regulatory region approximately 14 kb upstream of the lactase *LCT* gene, with different variants predominating in different populations. 13910C>T is particularly frequent in the populations of northern Europe and India, −13915T>G in the Near East, and other alleles in various East African and Sahelian pastoralist populations.^23^^,^^24^^,^^25^ However, some populations, such as those living across the Eurasian Steppe and in Mongolia, do not possess any of these alleles, yet consume large amounts of milk annually.^1^^,^^3^^,^^5^^,^^18^^,^^26^ Nevertheless, fundamental questions regarding the origins of these alleles, their spatiotemporal selective dynamics, which seem to be a more complex process than previously thought,^16^ and their prevalence in under-characterized regions, remain unanswered.

Ancient DNA studies have demonstrated the absence or low frequency of these LP alleles during the Neolithic period,^16^^,^^27^^,^^28^ suggesting that dairying practices developed largely in lactase non-persistent communities. This temporal mismatch between cultural and genetic evidence suggests that early consumers of dairy likely relied on fermentation and other processing techniques to reduce lactose content and mitigate intolerance symptoms.^29^ One of the earliest appearances of −13910C>T is reported in a Chalcolithic individual recovered from Eastern Europe, dated to 5,960 BP,^30^^,^^31^ though LP did not reach significant frequencies in Europe until after the Bronze Age.^32^ Although this increase has often been linked to the migration of populations from the Pontic-Caspian steppe around 5,000 years ago,^33^ this attribution is now debated.^34^^,^^35^ Recent findings highlight that many earlier studies have relied on imputation to detect the LP allele, an approach now considered unreliable when ancestral haplotypes lacking the T allele for rs4988235 are present in ancient DNA samples.^34^^,^^36^ Moreover, other LP-associated alleles are underrepresented or absent in current reference panels, further limiting the accuracy of imputation. Additionally, contamination of ancient samples presents a significant challenge, as does the absence of stringent filtering protocols. This is particularly problematic given that the rs4988235 SNP involves a C>T transition, which is characteristic of ancient DNA damage patterns. Therefore, rigorous and direct screening approaches are essential for accurate inference of LP in ancient samples.

Here, we integrate newly produced proteomic data with previously published genetic evidence to investigate the history of milk consumption and lactose digestion in Armenia. We report evidence of dairy consumption in ancient populations through liquid chromatography-tandem mass spectrometry (LC-MS/MS) analysis of dental calculus from individuals dating from the Late Neolithic to the Middle Ages (Figure 1). In parallel, we genetically screened available published genome-wide and whole-genome data on modern individuals of Armenian origin for SNPs associated with LP. We also screened published ancient DNA data from the region for LP alleles. The Armenian population has demonstrated a high level of regional genetic continuity for well over 6,000 years^37^ and, unlike many other parts of Eurasia, has not been substantially affected by major migrations, aside from a genetic input during the Late Bronze/Iron Age from a source related to Neolithic Levantine farmers. This makes it a compelling population in which to trace the temporal dynamics of this major cultural and nutritional adaptation through time. With this demographic context, our integrative approach allows us to reconstruct patterns of milk use over time and evaluate the extent to which cultural and genetic adaptations to dairying co-evolved in this key yet understudied region.

**Figure 1:**
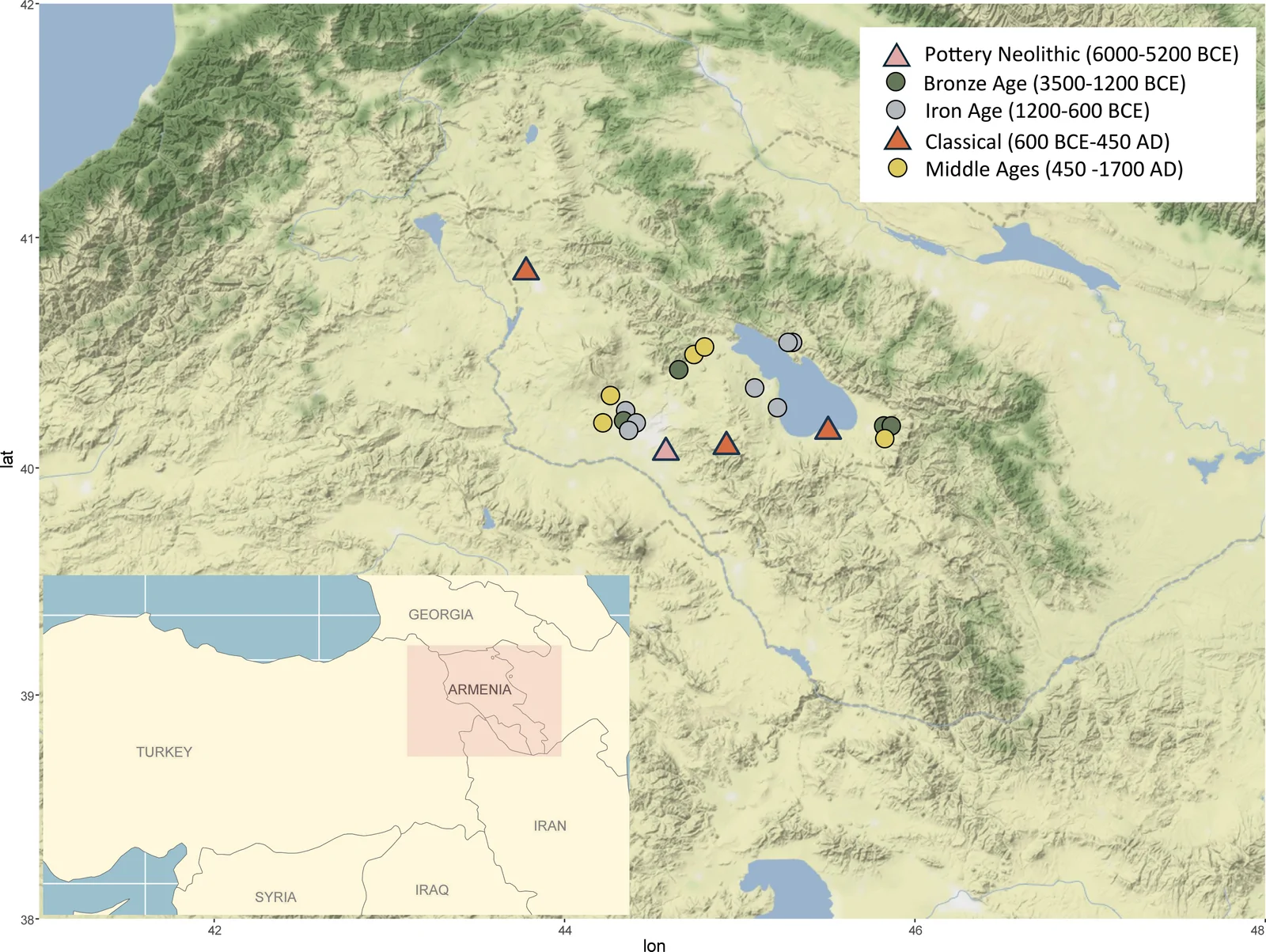
Locations of archaeological sites examined in the present study, spanning from the Late Neolithic to the Middle Ages (site descriptions are presented in Table S1)

## Results

### Dental calculus palaeoproteomic analysis

We performed a palaeoproteomic analysis of dental calculus from 77 individuals excavated from 22 archaeological sites, spanning the Late Neolithic to the Middle Ages (Figure 2 and Table S2). To evaluate protein preservation and detect potential contamination, both ancient dental calculus samples and laboratory controls were screened against the Oral Signature Screening Database (OSSD; see STAR Methods). Of the 77 analyzed samples, 60 (78%) exhibited well-preserved oral signatures, indicating their suitability for further analysis (Table S3).

**Figure 2:**
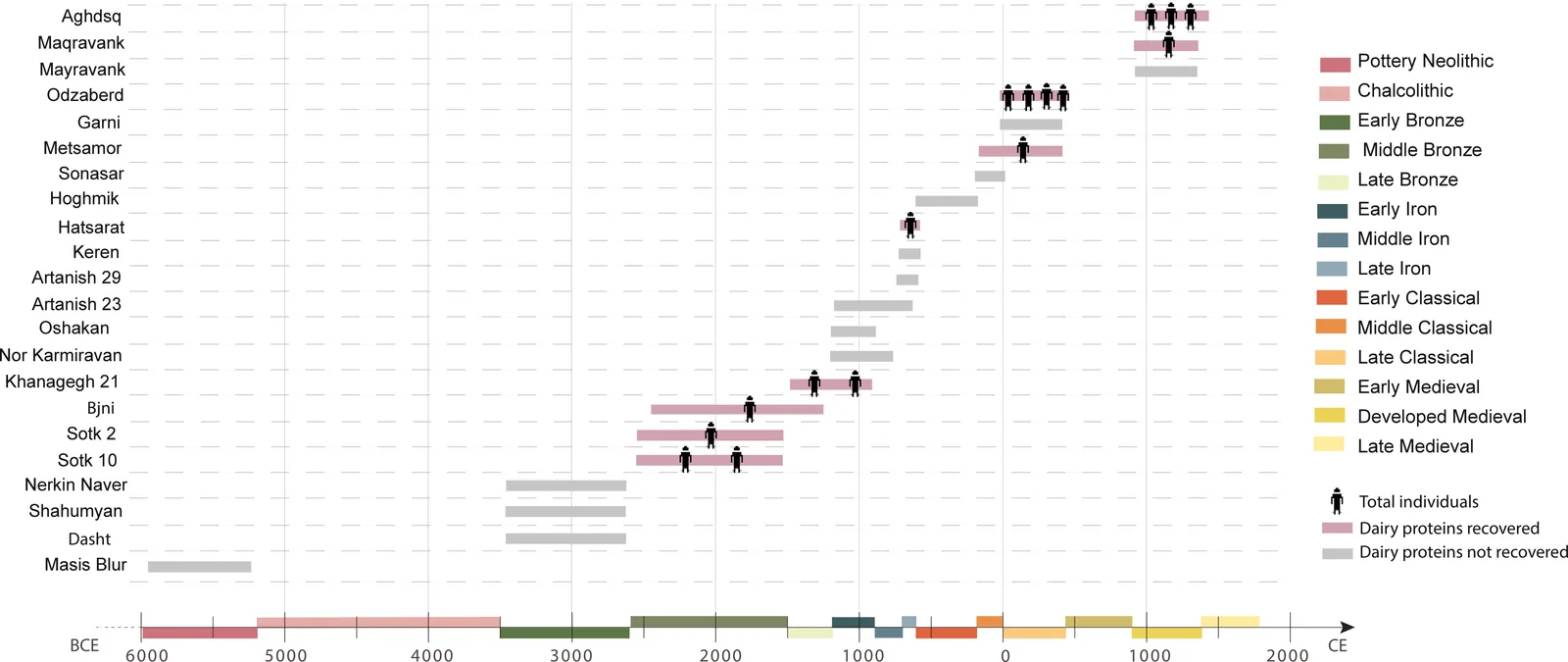
Summary of dairy protein recovery results for sites and individuals analyzed through palaeoproteomics Human symbols denote individuals whose calculus produced dairy peptides.

Of the 60 calculus samples that passed OSSD screening, dairy peptides were identified in 16 individuals from 9 sites (Table S3). The remaining 17 calculus samples did not yield dairy proteins. We report detected non-dairy proteins per individual in Table S4. The detected dairy peptides were predominantly derived from β-lactoglobulin (BLG), identified in 14 calculus samples (Figure 3), and β-casein, detected in 2 calculus samples. Most individuals with positive dairy protein results exhibited multiple peptide spectral matches to ruminant dairy proteins (Table S5). While some milk peptides were specific to only higher taxonomic levels (Pecora infraorder: *n* = 22 peptide spectrum matches [PSMs]; Bovidae family, *n* = 2; Caprinae subfamily: *n* = 6), others allowed for more refined taxonomic classification. In particular, peptides were assigned to milk from sheep (*Ovis*, *n* = 4), goat (*Capra*, *n* = 7), and/or cattle (*Bos*/Bovinae, *n* = 4). Due to ambiguity of deamidation, some of the detected milk peptides consisted of sequences that are indistinguishable between sheep and cattle, suggesting the consumption of milk from either species (*Ovis*/Bovinae, *n* = 55).^3^^,^^14^

**Figure 3:**
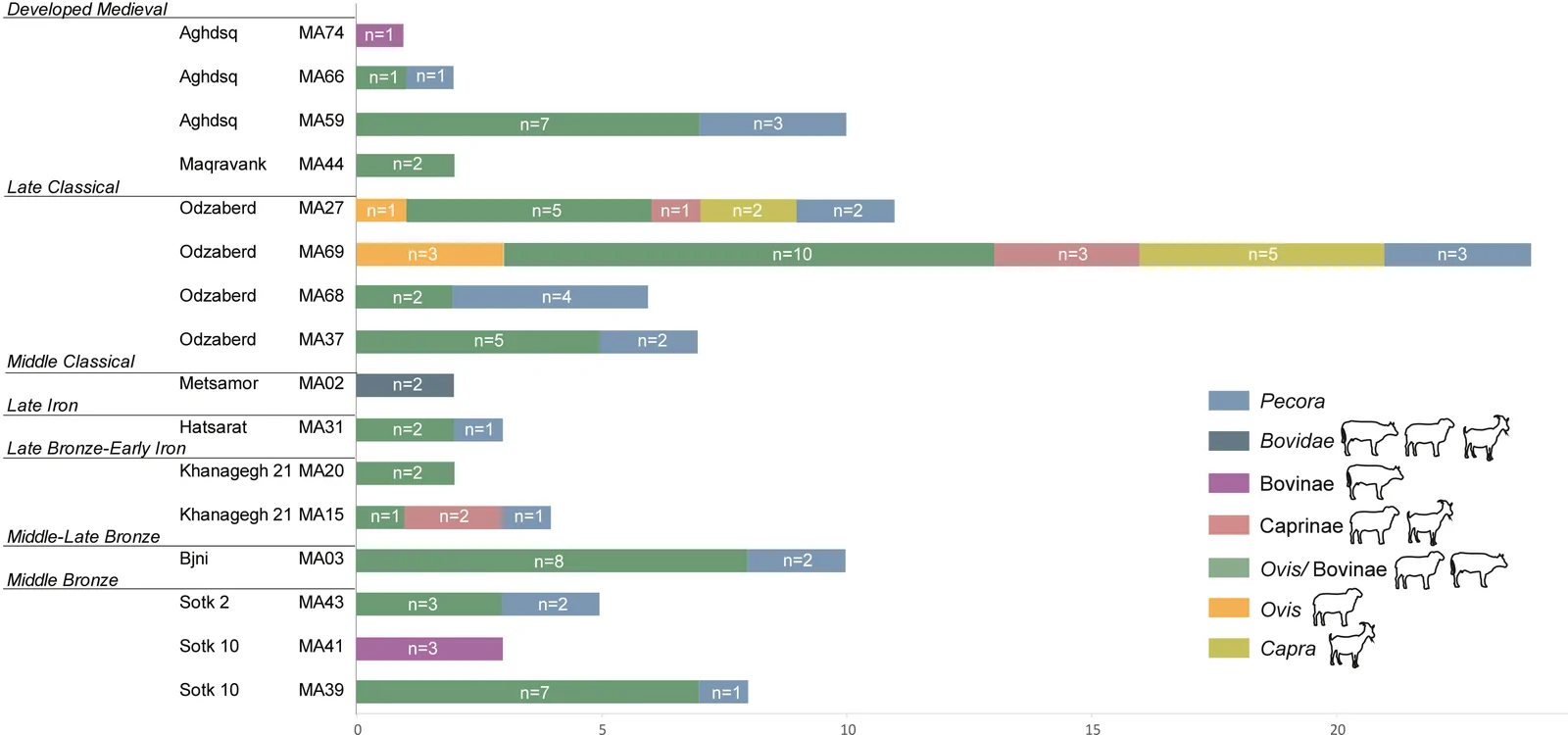
Taxonomic specificity of milk protein spectral matches for each individual with positive dairy results

The presence and distribution of recovered milk peptides fluctuates across different periods. The earliest individual in our study, MA100, an adult male between the ages of 45 and 60, excavated from the Late Neolithic site of Masis Blur, exhibited well-preserved oral proteins but showed no direct evidence of milk consumption. We also recovered a small amount of non-dairy-related peptides, which are detailed in the supplemental information (Table S5).

Similarly, among the nine Early Bronze Age individuals analyzed, four displayed well-preserved oral proteomes, yet none contained detectable dairy proteins. The earliest direct evidence of milk consumption in our dataset comes from the Middle Bronze Age, with all four individuals analyzed exhibiting milk proteins. Specifically, individuals MA39, MA43, and MA03 from the Sotk-10 site preserved BLG peptides specific to Pecora and *Ovis*/Bovinae, while analysis of an individual MA41 from the same site revealed peptides specific to cattle.

The Late Bronze–Early Iron Age assemblage includes dental calculus from six individuals, with five specimens exhibiting good preservation of the ancient oral proteome. Two individuals from the Kanagegh-2 site yielded milk peptides: MA15 contained BLG peptides assignable to Pecora, *Ovis*/Bovinae, and Caprinae, whereas MA20 displayed peptides can be attributed to *Ovis*/Bovinae. Among the ten individuals assigned to the Middle to Late Iron Age, six passed OSSD screening, but only one individual (MA31), from the Hatsarat site, exhibited evidence of milk consumption, with BLG peptides specific to Pecora and *Ovis*/Bovinae.

In the Middle Classical period, seven individuals were analyzed, of whom only one did not preserve dairy proteins; of these, one individual (MA02), from the Metsamor site, exhibited β-casein peptides specific to Bovidae. The Late Classical period is represented by ten individuals, eight of whom exhibited a well-preserved oral proteome. Of these, four individuals from Odzaberd had evidence of milk proteins: a male aged 50–55 years (MA37) and a female aged 35–45 years (MA68) displayed BLG peptides assignable to Pecora and *Ovis*/Bovinae, while a male aged 14–17 years (MA69) and a male aged 35–45 years (MA27) provided direct evidence of milk consumption from multiple taxa, with BLG peptides unique to both sheep and goat, as well as *Ovis*/Bovinae. The Medieval period includes 31 individuals, with only two failing the OSSD screening. Of the remaining 29 individuals, only four contained detectable milk proteins: MA44 exhibited BLG peptides matching *Ovis*/Bovinae, MA59 and MA66 displayed BLG peptides specific to Pecora and *Ovis*/Bovinae, and MA74 preserved β-casein peptides specific to Bovinae.

### Allele distribution in modern and ancient populations in Armenia

We explored the presence of SNPs associated with LP in 158 modern Armenian individuals. We found a modest frequency (∼6%–8%) for the 13910C>T variant (Table S6), which is the most commonly observed mutation in European populations. This variant is found in a high frequency in India, northwestern Africa, and the western African Sahel but is generally not prevalent in most East African and Middle Eastern populations. Interestingly, the other four variants, −13915T>G, −14010G>C, −13907C>G, and −14009T>G, were absent in the Armenian samples we analyzed (Figure S1 and Table S6). This pattern is noteworthy given Armenia’s geographical position at the crossroads between Europe and the Middle East, with the latter region having the −13915T>G allele as the most prevalent variant associated with LP. The 13910C>T locus showed no significant deviation from Hardy-Weinberg equilibrium in the full-genome dataset representing the pooled Armenian population, which could be explained by the relative homogeneity of Armenian populations from the different regions within the Armenian highlands.

We next screened for the presence of LP-associated variants in all published ancient samples from Armenia. Following imputation, nine samples showed positive results for the derived allele of rs4988235 (Tables S6 and S7). To confirm these results, we directly screened reads covering this position using stringent filtering criteria. While this position was not covered in most low-coverage ancient samples, we successfully confirmed the presence of the derived allele in a sample from Harzhis, dating to the Late Urartian period (680–550 BCE). The results for the other eight samples, including Early Bronze Age specimens from Armenia (2,600–2,300 BCE), require cautious interpretation, as they could represent false positives from imputation. In this scenario, they could have carried haplotypes that have ancestral rs4988235 genotypes but are similar to that on which the −13910C>T variant arose.^34^^,^^36^

We then aimed to directly check reads for the previously reported oldest case of the derived LP allele in a sample from Ukraine (I6561), dated to ∼4,000 BCE.^31^ With our stringent filtering approach, the sample yielded only a single sequence read with the derived allele, and given the partial-uracil-DNA glycosylase [UDG] library treatment method, we could not reject the possibility that this could be due to error. We also screened recently reported samples from Patterson et al. 2022,^38^ and the oldest sample we confirmed to carry the derived allele was an individual from the Czech Republic (I7953), dated to 3,000–2,000 BCE. Additionally, we screened samples from the neighboring regions, confirming the presence of the derived allele in a Middle Bronze Age individual from the Northern Caucasus (Tables S6 and S7).

## Discussion

Our palaeoproteomic findings shed light on the dynamics and specificities of human subsistence strategies in Armenia through time, particularly regarding the emergence, intensification, and diversification of milk consumption. On the basis of milk proteins identified in the dental calculus of 15 individuals from 9 sites, we demonstrate that milk products were consumed in the region by at least the 2nd millennium BCE (Middle Bronze Age), with cattle and, possibly, sheep (indicated by non-specific bovid milk peptides, which could derive from either sheep or cattle) serving as the primary sources of dairy products in these early dairying cultures. We find a continuity of cattle and/or sheep milk consumption in the region from the Middle Bronze Age through to the Medieval period. Over time, both the intensity of milk consumption and the diversity of exploited animals increased, with the first direct evidence of goat milk appearing in the Classical period. It should be noted, however, that the absence of dairy proteins in the individuals studied does not necessarily rule out milk consumption in a given period, as this may reflect sampling bias, small sample size, or dietary choices across different populations. It is noteworthy that milk proteins in dental calculus were predominantly recovered from high-altitude sites situated above 1,500 m asl. This pattern is particularly prominent in prehistoric occupations (Neolithic to Iron Age): of the 19 individuals with confirmed protein preservation, 7 yielded dairy peptides (all recovered from sites situated above 1,600 m asl), while 6 individuals from lowland sites with confirmed protein preservation showed no milk signal (Table 1). This pattern may reflect milk consumption as an adaptive strategy to cope with the harsh environmental conditions of highland regions, a hypothesis that requires further testing with larger sample sizes. A similar trend was observed in a study of milk residues in ceramic vessels^39^ from the Late Bronze Age, where milk use appeared to be more common in upland areas. This pattern was similarly seen in ancient Tibet, where milk consumption was only recovered from individuals in the high-latitude occupation sites, above the area where crop production was impossible due to environmental constraints.^2^

**Table 1:** Archaeological sites included in the palaeoproteomic analysis, showing site elevation, total number of individuals analyzed, number of individuals yielding identifiable proteins, and number of individuals with detected dairy peptides

Period	Site	Elevation (m asl)	Total individuals	Proteome preserved	Dairy peptides
Late Neolithic	Masis Blur	869	1	1	–
Early Bronze	Dasht	947	2	1	–
	Shahumyan	941	6	2	–
	Nerkin Naver	1,080	1	–	–
Middle Bronze	Sotk 10	2,100	2	2	2
	Sotk 2	2,101	1	1	1
Late Bronze	Bjni	1,620	1	1	1
Late Bronze-Iron	Khanagegh 21	1,940	5	4	2
Early Iron	Artanish 23	1,927	2	1	–
	Nor Karmiravan	460	1	1	–
Iron	Oshakan	1,090	1	–	–
	Hatsarat	1,915	3	2	1
	Keren	570	1	1	–
	Artanish 29	1,935	1	1	–
Late Iron	Artanish 23	1,927	2	1	–
Classical	Garni	1,380	2	1	–
	Hoghmik	2,000	2	1	–
	Metsamor	850	3	2	1
	Odzaberd	1,921	8	7	4
	Sonasar	1,350	1	1	–
Middle Ages	Aghtsq	1,230	24	23	3
	Makravank	1,783	3	3	1
	Mayravank	1,977	4	3	–

At the same time, the relationship between elevation and dairy consumption is not straightforward. Seven individuals from high-altitude sites passed the OSSD threshold yet did not yield dairy proteins. Notably, these individuals date from the Iron Age to the Middle Ages, a time frame during which milk proteins also begin to appear at lower-elevation sites. This suggests that elevation alone cannot explain the mosaic of dietary preferences through time. Instead, the pattern points to a more complex and temporally dynamic picture in which dairying practices and dietary preferences varied across both landscapes and time periods.

When contextualized with existing regional data,^18^^,^^39^ our results contribute to a more comprehensive understanding of the development and role of dairying in the South Caucasus as a whole. Early evidence for animal husbandry and food-producing economies in the South Caucasus dates to the Pottery Neolithic (6,000–5,500/5,200 BCE), a period characterized by extensive interactions between populations in eastern Anatolia, the South Caucasus, and the Levant.^40^^,^^41^ These close contacts may have facilitated the spread of animal husbandry as well as the transmission of dairying knowledge and milk consumption practices. Communities associated with the Neolithic Aratashen-Shulaveri-Shomutepe tradition are thought to have lived in lowland permanent settlements with fully sedentary lifeways, subsisting primarily on agriculture and animal husbandry.^42^^,^^43^^,^^44^^,^^45^ While some Neolithic zooarchaeological assemblages (e.g., Masis Blur and Aratashen) indicate caprine exploitation for secondary products, particularly dairy,^46^^,^^47^ no direct evidence for milk consumption has been documented for this period. This absence may reflect preservation biases or the limitations of small sample sizes, though it is also possible that, despite the full domestication and management of ruminants by the Late Neolithic, the practice of dairy consumption was not yet widespread in the region. If milk consumption was infrequent and comprised only a small percentage of the overall diet, it may be that milk proteins did not incorporate into the calculus biofilm. It must be noted that the absence of milk proteins does not equal an absolute lack of consumption.

The first direct evidence of milk consumption in the South Caucasus comes from a Chalcolithic individual at the Alkhantepe site, dated to ca. 3,700 BCE, where cattle milk proteins were identified.^18^ Similarly, the earliest evidence of milk consumption in the North Caucasus comes from the progress 2 site, dated to 4,338–4,074 BCE, where sheep milk consumption was detected.^18^ These results align with abundant zooarchaeological data from the region, where kill-off profiles and birth seasonality patterns suggest that early Chalcolithic pastoralists deliberately managed their herds to optimize access to milk, meat, and fiber.^48^^,^^49^^,^^50^^,^^51^^,^^52^

During the Early Bronze Age (3,500/3,350–2,500 BCE), the economic importance of pastoralism increased in the South Caucasus, where a unique cultural tradition, named the Kura-Araxes, grew out of Chalcolithic interactions in the mid-4th millennium BCE. These were non-hierarchical sedentary communities that practiced farming and herding.^53^^,^^54^^,^^55^ None of the Early Bronze Age individuals associated with the Kura-Araxes cultural complex, recovered from three archaeological sites and analyzed in our study, yielded milk proteins. This is in line with zooarchaeological records from the region, as kill-off patterns of regional sites indicate that sheep and goat were mainly exploited for meat consumption.^56^^,^^57^^,^^58^ Nevertheless, the absence of milk proteins is somewhat surprising given lipid residue analyses of Kura-Araxes ceramic vessels from seven Armenian sites, which revealed traces of ruminant milk, though at low levels, alongside a stronger dietary emphasis on meat.^39^ Similarly, recent biomolecular analyses of pottery from Qaraçinar settlement in Azerbaijan reveal the central role of dairy products and ruminant carcass fats in Kura-Araxes cuisine.^59^ While lipid profiles cannot identify the specific animal source, zooarchaeological data from the region point to a predominance of cattle and caprines, comprising over 50% of the identified faunal remains (number of identified specimens).^56^^,^^57^^,^^58^^,^^60^^,^^61^ Notably, the use of ceramic vessels for processing dairy products appears to have been more prevalent at highland settlements located above 1,000 m compared with lowland areas,^39^ suggesting differentiated management of animal resources across this cultural province. Although milk consumption clearly intensified from the Early Bronze Age onward, this cultural evolution proceeded without corresponding genetic adaptations, as LP-associated variants did not reach widespread distribution in local populations. While we could not directly confirm their presence in these early periods, we can reasonably infer that the ancestral haplotype on which the 13910C>T variant subsequently arose was already present in the region from at least the Early Bronze Age, albeit at relatively low frequencies.

During the Middle Bronze Age (2,500–1,550 BCE), inhabitants of Kura-Araxes communities appear to have shifted away from settled village life in favor of more mobile ways of living, likely sustained by mobile pastoralism.^62^^,^^63^^,^^64^^,^^65^^,^^66^ This transition was accompanied by significant changes in social organization, including the earliest clear evidence of social inequality, as reflected in the emergence of large burial kurgans across the region.^53^^,^^67^^,^^68^^,^^69^^,^^70^ Our results show a reliance on dairy technology for subsistence for these mobile pastoralists, confirmed at two highland sites along the mountainous shores of Lake Sevan (Sotk 2 and Sotk 10, situated at ca. 2,000 m asl), with cattle milk proteins specifically identified at Sotk-10. In contrast, a previously published analysis of an individual from the Hatsarat site, also located in the Sevan Basin, revealed evidence of sheep milk consumption.^71^ Meanwhile, proteomic analysis of material from the Middle Bronze Age site of Qızqala, in the Aras River Valley of Nakhchivan, detected the presence of goat milk proteins.^18^ These variations likely reflect ecological adaptations, with environmental conditions appearing to have shaped livestock management strategies. Goats, being more resilient to arid climates and scarce water, were favored in the dry lowlands of Nakhchivan, while the well-watered, pasture-rich Sevan Basin supported cattle and sheep herding.

After nearly a millennium of more mobile lifeways, communities of the Middle Bronze Age chose to establish more permanent agropastoral settlements in the Late Bronze Age (ca. 1,500–1,150 BCE), but now organized in the first complex territorial polities in the region, administered from hilltop fortresses.^65^^,^^67^^,^^68^^,^^72^^,^^73^ In our dataset, evidence of ruminant milk consumption persists from the Late Bronze, aligning with the period associated with the Lchashen-Metsamor cultural tradition. This includes a male individual aged 25–30 years, recovered from the high-altitude Kanagegh site (1,970 m asl) that showed proteomic evidence of caprine milk consumption. Additionally, a female individual, aged 30–40 years, recovered from another high-altitude site, Bjni (1,566 m asl), revealed evidence of sheep or cattle milk consumption.

During the Iron Age, evidence for milk consumption became more substantial and geographically diverse. An Early Iron Age individual from the Goytepe site in Azerbaijan revealed the presence of sheep milk proteins.^18^ Notably, although evidence of horse milking has been identified in Early Iron Age remains from the North Caucasus,^18^ associated with pre-Scythian groups repopulating the steppe, no comparable evidence exists for horse milk consumption in the South Caucasus. This absence may reflect a sampling bias; however, it could also indicate that horse milking was a steppe-specific adaptation practiced by mobile horse-reliant groups. Later in the Iron Age, between the 9th and 7th centuries BCE, the Kingdom of Urartu emerged in the basin of Lake Van and expanded its influence across what is now the South Caucasus. Urartu’s animal economy relied on sheep, goats, and cattle, with horses playing a key role in this militarized society.^74^^,^^75^ In our study, ruminant milk was detected in the Late Iron Age Urartian Hatsarat burial (1,990 m asl); however, the milk proteins consisted of non-specific bovid peptides, indicating either sheep or cattle. Significantly, our earliest confirmed presence of the rs4988235 variant in Armenia also dates to approximately 3,000 years ago, corresponding to this Late Urartian period. While the precise origin of the allele remains unknown, its confirmed appearance post-dates the documented Late Bronze/Iron Age genetic influx in the region,^37^ suggesting that it may reflect introduction through this or related population interactions.

For the Late Classical period, when the kingdom of Armenian Arsacids came to power in Armenia, our dataset reveals a notable shift in dairying strategies, marked by the exploitation of a broader range of livestock species. Among eight individuals analyzed from the Odzaberd site (1,963 m asl), two exhibited a diversified milk protein profile, including evidence of sheep, goat, and cattle milk consumption. The consumption of milk from multiple species may indicate the wealth or trade connections of the studied individuals; however, in the South Caucasus, mixed herding practices involving the concurrent management of cattle, sheep, and goats were common, reflecting a broader regional strategy of diversified livestock exploitation. Notably, this represents the earliest evidence of goat milk consumption within our assemblage. The first appearance of goat milk in the highlands of Armenia may indicate a diversification in dairy exploitation strategies. However, this pattern could also reflect sampling bias and requires further investigation.

Lastly, we present the only regional dataset documenting milk consumption during the Medieval period. In the Developed Medieval period, our findings suggest a potential correlation between social status and milk consumption. Of the 24 individuals analyzed from the Aghtsg burial complex (1,230 m asl), only two showed evidence of sheep or cattle milk proteins. Notably, both individuals were recovered from sarcophagi, indicating their elevated social status. The detection of dairy proteins exclusively in high-status individuals may indicate preferential access to milk products among the elite in this period. At the same time, we cannot exclude the possibility of better molecular preservation in sarcophagi, as these remains are less exposed to sunlight, rain, soil acidity, and other diagenetic and taphonomic processes. These findings underscore the need for further research into the interplay between social hierarchy and subsistence in the Medieval period in the Southern Caucasus.

To complement our historical analysis, we also investigated the prevalence of LP alleles in the modern Armenian population. Previous studies have indicated two controversial results, one suggesting a very low frequency of 2.7%^76^ and the other claiming 18%.^23^ Here, we aimed to readdress the question of the prevalence of LP-associated variants in the Armenian population, using recently published genetic datasets. Interestingly, we found an ∼8% prevalence of the European LP allele in a recently published whole-genome dataset on modern Armenians,^37^^,^^77^ falling between the previously reported estimates, and ∼6% when combined with published genotyping array data.^37^^,^^78^ We additionally report a complete absence of the predominant Middle Eastern LP variant in the population. We speculate that the relatively low frequency of LP-associated variants compared with many other populations may reflect the predominant use of fermented dairy products such as *matsun*, which contain reduced lactose levels. While there is currently no evidence for unidentified LP variants in Armenians, a formal genome-wide association study (GWAS) focused on this population would be required to formally rule out this possibility.

This study represents the first multidisciplinary approach of its kind that combines genetic and palaeoproteomic evidence in examining dairy usage for a single region across a wide temporal framework. By tracing over 6,000 years of dairy consumption in Armenia, it demonstrates that, despite early and intensive dairying in the region, genetic adaptation to dairy consumption through LP arose relatively late and, furthermore, never became widespread, remaining relatively rare, even among contemporary Armenians despite the fact that dairy continues to be important in Armenian cuisine. These findings demonstrate that cultural and dietary strategies—such as fermentation, cheese-making, and/or mixing milk with other foods to reduce lactose—rather than genetic adaptation, were critical in enabling regional populations to sustain and benefit from dairying. Key questions, nonetheless, remain regarding the origins of dairying and whether milk consumption served as the primary selective pressure driving the evolution of LP, or if other factors contributed to this genetic adaptation.

Further research incorporating larger sample sizes and a greater number of sites, not only from Armenia but also from adjacent regions, is needed to better understand the history of milk consumption in the area. Additionally, complementary proxies such as ceramic residue analysis and stable isotope analysis of human dental and bone remains would provide a more comprehensive understanding of dietary preferences in the region. Ancient DNA studies with direct screening evidence are required to shed light on the question of origins and spread of LP alleles in the region.

### Limitations of the study

This study is constrained by the availability and preservation of archaeological and genetic material. Human remains with preserved dental calculus are particularly scarce for the earliest periods, with only one Late Neolithic individual and no Chalcolithic individuals available from Armenia, limiting our ability to assess milk consumption during these periods. Biomolecular preservation also varied among samples, and the absence of proteins cannot necessarily be taken as evidence that milk was not consumed. Similarly, the ancient genetic dataset is limited by the availability of published genomes and insufficient genomic coverage, particularly for early periods, preventing direct assessment of LP-associated variants in many individuals. We relied on imputation to assess LP-associated genotypes, but non-European LP-associated alleles are underrepresented or absent in current reference panels. Where sequencing depth allowed, we cross-checked imputed genotypes at rs4988235 against directly observed reads; for individuals lacking sufficient coverage at this position, genotypes rely on imputation alone and should be interpreted with caution. These limitations mean that the observed chronological and geographical patterns should be interpreted cautiously and highlight the need for larger, better-represented archaeological and genomic datasets.

## Resource availability

### Lead contact

Requests for further information and resources should be directed to and will be fulfilled by the lead contact, Mariya Antonosyan (antonosyan@gea.mpg.de).

### Materials availability

• This study did not generate new unique reagents
• This paper does not report original code.
• All data generated or analyzed during this study are included in this paper and its supplemental information files.

### Data and code availability

• All data generated in this study are available in the main text or supplemental information section of this article and listed in the key resources table.
• This paper does not report original code.
• Any additional information required to reanalyze the data reported in this paper is available from the lead contact upon request.

## Acknowledgments

The research was funded by the 10.13039/501100004189Max Planck Society. L.Y., Z.K., and S.M. were supported by the Higher Education and Science Committee, MESCS, Armenia (projects #21AG-1F025).

## Author contributions

Conceptualization, M.A.; investigation, M.A. and A.H.; formal analysis, M.A., A.H., and L.M.C.; writing – original draft, M.A. and A.H.; writing – review & editing, M.A., A.H., P.R., S.M., Hasmik Simonyan, A.B., L.A., A.P., R.B., N.Y., A. Gnuni, H.A., M.B., A. Gabrielyan, N.S., A. Grigoryan, Hakob Simonyan, M.S., B.V., K.M.-O., A. Gyonjyan, Z.K., J.I., L.Y., A.M., D.G.B., N.B., and S.W.; visualization, M.A., A.H., and S.M.; resources, P.R., J.I., and N.B.; archaeological data acquisition, Hasmik Simonyan, A.B., L.A., A.P., R.B., N.Y., A. Gnuni, H.A., M.B., A. Gabrielyan, N.S., A. Grigoryan, Hakob Simonyan, M.S., and K.M.-O.; project administration, M.A.; supervision, S.W.

## Declaration of interests

The authors declare no competing interests.

## STAR★Methods

### Key resources table

**Table:** 

REAGENT or RESOURCE	SOURCE	IDENTIFIER
**Deposited data**		

All LC-MS/MS data files (.raw), MGF files (.mgf), and resulting Mascot search files (.dat) have been deposited at ProteomeXchange and are publicly available as of the date of publication.	https://www.proteomexchange.org/	ProteomeXchange: PXD067611 and PXD067613

**Software and algorithms**		

Mascot, Matrix Science version 2.7.0.1	N/A	https://www.matrixscience.com/
MS-MARGE: Mascot Report Generator	N/A	https://bitbucket.org/rwhagan/ms-marge/src/master/
ProteoWizard package v.3.0.11781	N/A	https://proteowizard.sourceforge.io/
plink2	Purcell et al.^79^	N/A
GATK version 4.3.0.0	McKenna et al.^80^	N/A
BWA version 0.7.17-r1188	Li^81^	N/A
Cutadapt version 3.5	Martin^82^	N/A
AdapterRemoval version 2.2.2	Schubert et al.^83^	N/A
Picard version 2.22.1	N/A	http://broadinstitute.github.io/picard
Samtools version 1.7	Danecek et al.^84^	N/A
GLIMPSE version 1.1.0	Rubinacci et al.^85^	N/A

**Other**		

Recorded dairy and non-dairy peptides and allele distribution in modern and ancient populations are available in Supplementary Tables	This paper	Supplementary data

### Method details

#### Sample collection

To examine ancient dairy consumption history, we analyzed ancient proteins extracted from the dental calculus of 76 individuals excavated from 22 archaeological sites across Armenia spanning the Late Neolithic to Medieval period (Figure 1). The detailed archaeological contexts for each site are described in Table S1. The list of individuals, including their age and sex profiles, is presented in Table S2.

Dental calculus samples were collected in a dedicated clean laboratory at the Institute of Molecular Biology, NAS Armenia, under controlled conditions to reduce the risk of contamination. Nitrile gloves, surgical masks, and hair nets were worn throughout the sampling process to minimise the risk of modern contamination. Calculus was carefully detached from teeth using dental scalpels. To prevent cross-contamination between individuals, scalpels, gloves, and work surfaces were thoroughly cleaned with 75% ethanol between each sampling. The calculus samples were immediately transferred into sterile 1.5 mL Eppendorf tubes and stored until protein extraction, carried out at the dedicated Palaeoproteomics Laboratory at the Max Planck Institute for Geoanthropology (formerly MPI for the Science of Human History). Before extraction, 5–10 mg of calculus was weighed out in a microcentrifuge tube. Additionally, 2–3 mg of ancient sheep bone powder was prepared as a positive control.

#### Protein extraction

Samples were extracted using a single-pot, solid-phase enhanced sample preparation (SP3) modified for archaeological dental calculus samples.^78^^,^^86^^,^^87^^,^^88^ The full protocol can be found on protocols.io, DOI: https://dx.doi.org/10.17504/protocols.io.bfgrjjv6. Samples (including the positive control and extraction blank) were demineralised in 500 μL of 0.5 M Ethylenediaminetetraacetic acid (EDTA) for 4–7 days at room temperature. Following demineralisation, samples were centrifuged at 20,000 rcf for 10 min, and 400 μL of the supernatant was removed and retained. The remaining samples were denatured with 200 μL of 6 M guanidine hydrochloride (GuHCl), as well as reduced and alkylated 30 μL of 100 mM alkylated with chloroacetamide (CAA)/100 mM Tris(2-carboxyethyl)phosphine (TCEP). Samples were then incubated at 99 °C for 10 min. Afterward, 20 μL of a 20 μg μL^−1^ 50/50 mixture of hydrophilic and hydrophobic SeraMag SpeedBeads was added to each sample. To increase protein–bead adhesion, 350 μL of 100% ethanol was then added to each tube. Samples were then placed on the Thermoshaker at 1,000 rpm, 24 °C for 5 min. Afterward, tubes were placed on a magnetic rack, which induced the migration of beads (carrying the proteins) to the wall of the tube. The sample/ethanol solution was removed and retained. The beads were then washed with 200 μL 80% ethanol. Upon washing, 100 μL of 100 mM ammonium bicarbonate was added to each tube, as well as 0.2 μg of trypsin to initiate enzymatic digestion. The samples were incubated on a Thermoshaker at 750 rpm, 37 °C for 10 min. After, they were resuspended and left to incubate overnight (approximately 18 h). Following digestion, the tubes were centrifuged at 20,000 rcf for 1 min and returned to the magnetic rack. The supernatant was transferred to clean tubes. To stop trypsin digestion, each sample was acidified using 5% trifluoroacetic acid (TFA). The acidified samples were then centrifuged at maximum speed for 5 min to pellet any residual non-proteinaceous material. Peptides were then desalted with C18 StageTips. Samples were sent on stage tips to the Functional Genomics Center Zürich, ETH/University of Zürich for LC-MS/MS analysis.

#### LC-MS/MS analysis

LC-MS/MS analysis was performed on a Q Exactive mass spectrometer (Thermo Scientific) equipped with a Digital PicoView source (New Objective) and coupled to a nanoAcquity UPLC (Waters). The solvent composition at the two channels was 0.1% formic acid for channel A and 0.1% formic acid, 99.9% ACN for channel B. The column temperature was maintained at 50 °C. For each sample, 4 μL of peptides were loaded on a commercial ACQUITY UPLC M-Class Symmetry C18 Trap column (100 Å, 5 μm, 180 μm × 20 mm, Waters) followed by ACQUITY UPLC M-Class HSS T3 column (100 Å, 1.8 μm, 75 μm × 250 mm, Waters). The peptides were eluted at a flow rate of 300 nL min−1 by a gradient from 8 to 22% B in 49 min and to 32% B in an additional 11 min. The column was subsequently washed by increasing to 95% B and holding 95% B for 5 min before re-establishing the initial conditions.

The mass spectrometer was operated in data-dependent mode, acquiring a full-scan mass spectra (300–1,700 m/z) at a resolution of 70,000 at 200 m/z after accumulation to a target value of 3,000,000 and a maximum injection time of 110 ms followed by HCD fragmentation on the 12 most intense signals per cycle. HCD spectra were acquired at a resolution of 35,000 using a normalized collision energy of 25 and a maximum injection time of 110 ms. The automatic gain control was set to 50,000 ions. Charge state screening was enabled. Singly, unassigned and charge states higher than seven were rejected. Only precursors with intensity above 9,100 were selected for MS/MS measurement. Precursor masses previously selected for MS/MS measurement were excluded from further selection for 30 s and the exclusion window was set at 10 ppm. The samples were acquired using internal lock mass calibration on m/z 371.1012 and 445.1200.

### Quantification and statistical analysis

Generated raw LC-MS/MS spectra were converted to Mascot generic files using MSConvert from the ProteoWizard software package v.3.0.11781 by selecting the top 100 peaks from each spectrum. Each sample was searched using Mascot (Matrix Science; v.2.6.0) against the SwissProt database in combination with a custom curated dairy database consisting of 245 dairy livestock milk protein sequences.^3^ Mascot searches were conducted with a peptide mass tolerance of 10 ppm, with fragment mass tolerance of 0.01 Da and an allowance for monoisotopic mass shifts. Up to three missed cleavages were allowed. Carbamidomethyl of cystine (C) was set as a fixed modification, together with deamidation of asparagine (N) and glutamine (Q) and oxidation of methionine (M) as variable modifications.

To estimate the validity of peptide identifications and summarise the findings, the results were processed through an internally created tool, MS-MARGE,^89^ available for use via Bitbucket: https://bitbucket.org/rwhagan/ms-marge/src/master/. As input, MS-MARGE accepts a csv file exported from a Mascot MS/MS ion search against an amino acid database containing decoyed sequences with the Group Protein Families option turned off. MS-MARGE calculates the false discovery rate (FDR) at the PSM and protein level by comparing the number of forward sequence hits to the number of reverse sequence hits. For each sample, the protein false-discovery rate was targeted at under 5% and a peptide false-discovery rate at under 2%. A minimum of two individual peptide spectral matches were required for each specific protein identification, and only peptide spectral matches with an e-value of below 0.01 were accepted. To further investigate the taxonomic assignment, BLAST and Uniprot searches were performed on the identified peptides. Dietary peptides recovered per individual are reported in Table S5.

#### Proteome preservation assessment

Proteome preservation of each dental calculus sample was assessed using a curated Oral Signature Screening Database (OSSD).^5^^,^^14^^,^^90^ The OSSD contains common contamination proteins, including those encountered in the environment and laboratory, common bacteria found in the human microbiome, and human immune proteins that are regularly identified in the oral cavity. The screening approach is based on the premise that well-preserved ancient dental calculus retains a representative profile of oral microbiota and immune proteins typically present in saliva. Consequently, calculus samples exhibiting a strong oral proteomic signature—defined by meeting the OSSD threshold—are considered more likely to yield authentic insights into ancient dietary practices. To meet the OSSD criteria, a sample must contain a minimum of 15 identified OSSD proteins, with over 50% attributed to oral bacterial taxa and/or human inflammatory response proteins. Sample authenticity is further supported by the absence of dietary proteins in all positive (archaeological sheep bone with known proteome) and negative controls (extraction blank). Table S3 contains the overall count of OSSD proteins pulled from our filtered results, as well as the result of the oral microbiome protein identifiers. Table S4 reports proteins pulled from OSSD screening.

#### Genetic analyses: Modern data

We screened five lactase persistence-associated variants in the full genomes of 36 Armenians, each with all four grandparents (4 GP) originating from the same region within the Armenian highlands.^37^^,^^77^ In addition, we screened genotype data from 23 4 GP Armenian individuals^37^ and 99 non-4GP Armenians for the −13910C>T allele.^91^ The data has been processed as it is described in Hovhannisyan et al. 2025.^37^ plink2 was used to convert vcf files to the PLINK binary format, calculate allele frequency, and Hardy-Weinberg equilibrium for the given locus.^79^

#### Genetic analyses: Ancient data

We downloaded sequencing data for all previously published ancient samples from Armenia.^33^^,^^41^^,^^92^^,^^93^^,^^94^^,^^95^ When available, the data were downloaded and aligned from FASTQ files or, alternatively, realigned from provided BAM files. Adapter sequences were removed using Cutadapt 3.5^82^ and AdapterRemoval2.2.2^83^ for samples sequenced with single-end and paired-end technologies, respectively. The genomes were aligned to GRCh37 with decoy contigs (hs37d5) human reference genome using BWA aln^81^ with non-default parameters -l 16500, -n 0.02 and -o 2. Aligned reads were filtered for a minimum mapping quality of 25 using samtools version 1.7^84^ and for a minimum read length of 34 bp. Optical duplicates were removed using Picard Tools v2.22.1 MarkDuplicates http://broadinstitute.github.io/picard. Soft clipping was performed by reducing the base quality scores of the first and last two bases of each read to a Phred score of 2. For manual variant screening, we used GATK pileup –verbose,^80^ filtered for base quality 30. The two high-coverage Bronze Age individuals^95^ were genotyped with HaplotypeCaller in GATK. mapDamage was used to rescale base qualities.^96^ Modern and ancient DNA dataset used in the study are listed in Table S6.

#### Imputation

Biallelic variants and genotype likelihoods were called with “-mpileup” and “-call” in bcftools v1.1,^84^ respectively. In order to detect the haplotypes on which the LP allele arose or which potentially carry the allele, we imputed both captured and shotgun ancient genomes with GLIMPSE v1.0.1 using 1000 Genomes Project Phase 3 1000 Genomes Project Consortium.^97^ To avoid batch effects, imputation was conducted on each sample individually. Genomes were initially divided into 2 Mb windows with a 200 kb buffer using GLIMPSE_chunk,^85^ then imputed with GLIMPSE_phase and the resulting chunks were joined together using GLIMPSE_ligate. Variants were filtered based on genotype likelihood scores >0.99. Table S7 reports the presence of the rs4988235 derived allele in ancient DNA datasets.

## Supplemental information

Document S1. Figure S1

Table S1. Archaeological site descriptions

Table S2. Dental calculus remains analyzed in this study

Table S3. OSSD screening counts

Table S4. Peptides pulled from OSSD screening per individual

Table S5. Dietary peptides recovered per individual

Table S6. Modern and ancient DNA datasets used in the study

Table S7. Presence of the rs4988235-derived allele in ancient DNA datasets

## References

[1] Jeong C., Wilkin S., Amgalantugs T., Bouwman A.S., Taylor W.T.T., Hagan R.W., Bromage S., Tsolmon S., Trachsel C., Grossmann J. (2018). Bronze Age population dynamics and the rise of dairy pastoralism on the eastern Eurasian steppe. Proc. Natl. Acad. Sci. USA.

[2] Tang L., Wilkin S., Richter K.K., Bleasdale M., Fernandes R., He Y., Li S., Petraglia M., Scott A., Teoh F.K.Y. (2023). Paleoproteomic evidence reveals dairying supported prehistoric occupation of the highland Tibetan Plateau. Sci. Adv..

[3] Wilkin S., Ventresca Miller A., Taylor W.T.T., Miller B.K., Hagan R.W., Bleasdale M., Scott A., Gankhuyg S., Ramsøe A., Uliziibayar S. (2020). Dairy pastoralism sustained eastern Eurasian steppe populations for 5,000 years. Nat. Ecol. Evol..

[4] Bocquet-Appel J.-P. (2011). When the world's population took off: the springboard of the Neolithic Demographic Transition. Science.

[5] Wilkin S., Ventresca Miller A., Fernandes R., Spengler R., Taylor W.T.-T., Brown D.R., Reich D., Kennett D.J., Culleton B.J., Kunz L. (2021). Dairying enabled Early Bronze Age Yamnaya steppe expansions. Nature.

[6] Ventresca Miller A.R., Wilkin S., Hendy J., Turbat T., Batsukh D., Bayarkhuu N., Giscard P.-H., Bemmann J., Bayarsaikhan J., Miller B.K. (2022). The spread of herds and horses into the Altai: How livestock and dairying drove social complexity in Mongolia. PLoS One.

[7] Vigne J.-D., Bocquet-Appel J.-P., Bar-Yosef O. (2008). The Neolithic Demographic Transition and its Consequences.

[8] Vigne J.-D., Helmer D. (2007). Was milk a “secondary product” in the Old World Neolithisation process? Its role in the domestication of cattle, sheep and goats. Anthropozoologica.

[9] Vigne J.-D. (2011). The origins of animal domestication and husbandry: a major change in the history of humanity and the biosphere. C. R. Biol..

[10] Evershed R.P., Payne S., Sherratt A.G., Copley M.S., Coolidge J., Urem-Kotsu D., Kotsakis K., Ozdoğan M., Ozdoğan A.E., Nieuwenhuyse O. (2008). Earliest date for milk use in the Near East and southeastern Europe linked to cattle herding. Nature.

[11] Nieuwenhuyse O.P., Roffet-Salque M., Evershed R.P., Akkermans P.M.M.G., Russell A. (2015). Tracing pottery use and the emergence of secondary product exploitation through lipid residue analysis at Late Neolithic Tell Sabi Abyad (Syria). J. Archaeol. Sci..

[12] Hendy J., Colonese A.C., Franz I., Fernandes R., Fischer R., Orton D., Lucquin A., Spindler L., Anvari J., Stroud E. (2018). Ancient proteins from ceramic vessels at Çatalhöyük West reveal the hidden cuisine of early farmers. Nat. Commun..

[13] Dunne J., di Lernia S., Chłodnicki M., Kherbouche F., Evershed R.P. (2018). Timing and pace of dairying inception and animal husbandry practices across Holocene North Africa. Quat. Int..

[14] Bleasdale M., Richter K.K., Janzen A., Brown S., Scott A., Zech J., Wilkin S., Wang K., Schiffels S., Desideri J. (2021). Ancient proteins provide evidence of dairy consumption in eastern Africa. Nat. Commun..

[15] Debono Spiteri C., Gillis R.E., Roffet-Salque M., Castells Navarro L., Guilaine J., Manen C., Muntoni I.M., Saña Segui M., Urem-Kotsou D., Whelton H.L. (2016). Regional asynchronicity in dairy production and processing in early farming communities of the northern Mediterranean. Proc. Natl. Acad. Sci. USA.

[16] Evershed R.P., Davey Smith G., Roffet-Salque M., Timpson A., Diekmann Y., Lyon M.S., Cramp L.J.E., Casanova E., Smyth J., Whelton H.L. (2022). Dairying, diseases and the evolution of lactase persistence in Europe. Nature.

[17] Outram A.K., Kasparov A., Stear N.A., Varfolomeev V., Usmanova E., Evershed R.P. (2012). Patterns of pastoralism in later Bronze Age Kazakhstan: new evidence from faunal and lipid residue analyses. J. Archaeol. Sci..

[18] Scott A., Reinhold S., Hermes T., Kalmykov A.A., Belinskiy A., Buzhilova A., Berezina N., Kantorovich A.R., Maslov V.E., Guliyev F. (2022). Emergence and intensification of dairying in the Caucasus and Eurasian steppes. Nat. Ecol. Evol..

[19] Hirata M. (2020). Springer Geography.

[20] Afrikian E. (2012). Studies of lactic-acid bacteria in Armenia with emphasis on radioprotective properties. Environmentalist.

[21] Durham W.H. (1992).

[22] Tishkoff S.A., Reed F.A., Ranciaro A., Voight B.F., Babbitt C.C., Silverman J.S., Powell K., Mortensen H.M., Hirbo J.B., Osman M. (2007). Convergent adaptation of human lactase persistence in Africa and Europe. Nat. Genet..

[23] Liebert A., López S., Jones B.L., Montalva N., Gerbault P., Lau W., Thomas M.G., Bradman N., Maniatis N., Swallow D.M. (2017). World-wide distributions of lactase persistence alleles and the complex effects of recombination and selection. Hum. Genet..

[24] Ingram C.J.E., Mulcare C.A., Itan Y., Thomas M.G., Swallow D.M. (2009). Lactose digestion and the evolutionary genetics of lactase persistence. Hum. Genet..

[25] Gallego Romero I., Basu Mallick C., Liebert A., Crivellaro F., Chaubey G., Itan Y., Metspalu M., Eaaswarkhanth M., Pitchappan R., Villems R. (2012). Herders of Indian and European cattle share their predominant allele for lactase persistence. Mol. Biol. Evol..

[26] Jeong C., Wang K., Wilkin S., Taylor W.T.T., Miller B.K., Ulziibayar S., Stahl R., Chiovelli C., Bemmann J.H., Knolle F. (2020). A dynamic 6,000-year genetic history of Eurasia's Eastern Steppe. arXiv.

[27] Ségurel L., Bon C. (2017). On the evolution of lactase persistence in humans. Annu. Rev. Genomics Hum. Genet..

[28] Burger J., Kirchner M., Bramanti B., Haak W., Thomas M.G. (2007). Absence of the lactase-persistence-associated allele in early Neolithic Europeans. Proc. Natl. Acad. Sci. USA.

[29] Rosenstock E., Ebert J., Scheibner A. (2021). Cultured milk. Curr. Anthropol..

[30] Segurel L., Guarino-Vignon P., Marchi N., Lafosse S., Laurent R., Bon C., Fabre A., Hegay T., Heyer E. (2020). Why and when was lactase persistence selected for? Insights from Central Asian herders and ancient DNA. PLoS Biol..

[31] Mathieson I., Alpaslan-Roodenberg S., Posth C., Szécsényi-Nagy A., Rohland N., Mallick S., Olalde I., Broomandkhoshbacht N., Candilio F., Cheronet O. (2018). The genomic history of southeastern Europe. Nature.

[32] Mathieson I., Lazaridis I., Rohland N., Mallick S., Patterson N., Roodenberg S.A., Harney E., Stewardson K., Fernandes D., Novak M. (2015). Genome-wide patterns of selection in 230 ancient Eurasians. Nature.

[33] Allentoft M.E., Sikora M., Sjögren K.-G., Rasmussen S., Rasmussen M., Stenderup J., Damgaard P.B., Schroeder H., Ahlström T., Vinner L. (2015). Population genomics of Bronze Age Eurasia. Nature.

[34] Burger J., Link V., Blöcher J., Schulz A., Sell C., Pochon Z., Diekmann Y., Žegarac A., Hofmanová Z., Winkelbach L. (2020). Low prevalence of lactase persistence in Bronze Age Europe indicates ongoing strong selection over the last 3,000 years. Curr. Biol..

[35] Irving-Pease E.K., Refoyo-Martínez A., Barrie W., Ingason A., Pearson A., Fischer A., Sjögren K.-G., Halgren A.S., Macleod R., Demeter F. (2024). The selection landscape and genetic legacy of ancient Eurasians. Nature.

[36] Ali A.T., Liebert A., Lau W., Maniatis N., Swallow D.M. (2022). The hazards of genotype imputation in chromosomal regions under selection: A case study using the Lactase gene region. Ann. Hum. Genet..

[37] Hovhannisyan A., Delser P.M., Hakobyan A., Jones E.R., Schraiber J.G., Antonosyan M., Margaryan A., Xue Z., Jeon S., Bhak J. (2025). Demographic history and genetic variation of the Armenian population. Am. J. Hum. Genet..

[38] Patterson N., Isakov M., Booth T., Büster L., Fischer C.-E., Olalde I., Ringbauer H., Akbari A., Cheronet O., Bleasdale M. (2022). Large-scale migration into Britain during the Middle to Late Bronze Age. Nature.

[39] Manoukian N., Whelton H.L., Dunne J., Badalyan R., Smith A.T., Simonyan H., Rothman M.S., Bobokhyan A., Hovsepyan R., Avetisyan P. (2022). Diverse dietary practices across the Early Bronze Age “Kura-Araxes culture” in the South Caucasus. PLoS One.

[40] Skourtanioti E., Erdal Y.S., Frangipane M., Balossi Restelli F., Yener K.A., Pinnock F., Matthiae P., Özbal R., Schoop U.-D., Guliyev F. (2020). Genomic history of Neolithic to bronze age Anatolia, northern Levant, and Southern Caucasus. Cell.

[41] Lazaridis I., Alpaslan-Roodenberg S., Acar A., Açıkkol A., Agelarakis A., Aghikyan L., Akyüz U., Andreeva D., Andrijašević G., Antonović D. (2022). The genetic history of the Southern Arc: A bridge between West Asia and Europe. Science.

[42] Badalyan R., Chataigner C., Harutyunyan A., Badalyan R., Chataigner C., Harutyunyan A. (2022). Excavation seasons 2004–2015.

[43] Chataigner C., Badalyan R., Arimura M. (2014).

[44] Badalyan R., Lombard P., Chataigner C., Avetisyan P., Sagona A. (2004). A View from the Highlands: Archaeological Studies in Honour of Charles Burney.

[45] Martirosyan-Olshansky K., Areshian G.E., Avetisyan P.S., Hayrapetyan A. (2013). Masis blur: a late neolithic settlement in the Plain of Ararat, Armenia. Backdirt.

[46] Berthon R. (2014). Studies in Caucasian Archaeology.

[47] Bălăşescu A., Vila E., Radu V., Badalyan R., Chataigner C. (2010). Production animale et économie de subsistence au Néolithique dans la Plaine de l'Ararat (Arménie). Annales d'Université “Valahia” Târgovişte - Section d'Archéologie et d'Histoire.

[48] Antonosyan M., Maurer G., Mkrtchyan S., Boxleitner K., Saribekyan M., Hovhannisyan A., Furquim L., Stokes F., Davtyan R., Bobokhyan A. (2025). A biomolecular perspective on mobile pastoralism and its role in wider socioeconomic connections in the Chalcolithic South Caucasus. iScience.

[49] Palumbi G., Kalantaryan I., Balasescu A., Barge O., Chahoud J., Hovsepyan R., Meliksetian K., Avetisyan P., Chataigner C. (2021). Early pastoralism and natural resource management: recent research at Godedzor.On Salt, Copper and Gold: The Origins of Early Mining and Metallurgy in the Caucasu. C. Marro, T. Stöllner, eds., pp.

[50] Berthon R., Giblin J., Balasse M., Fiorillo D., Bellefroid É., Marro C., Stöllner T. (2021). On Salt, Copper and Gold.

[51] Decaix A., Martin L., Messager E., Chahoud J., Varoutsikos B., Mgeladze A., Gabunia M., Agapishvili T., Chataigner C. (2024). Subsistence economy in the South Caucasus during the Early Chalcolithic period: bioarchaeological analysis of Bavra Ablari rockshelter (Samtskhe-Javakheti region, Georgia). Front. Environ. Archaeol..

[52] Samei S., Hovhannisyan N., Gasparyan B. (2020). Economic and symbolic role of animals during the Late Chalcolithic period of Areni-1 Cave, Armenia. J. Archaeolog. Sci.: Rep..

[53] Kohl P.L. (2012).

[54] Sagona A. (2014). Rethinking the Kura-Araxes Genesis. Paleorient.

[55] Badalyan R.S. (2014). New data on the periodization and chronology of the Kura-Araxes culture in Armenia. Paleorient.

[56] Palumbi G. (2016).

[57] Piro J. (2008). Pastoral economies in Early Transcaucasian communities from the mid-4th to 3rd millennium BC. MOM Éditions.

[58] Badalyan R., Smith A., Lindsay I., Harutyunyan A., Greene A., Marshall M., Monahan B., R. H. (2015). A preliminary report on the 2008, 2010, and 2011 investigations of Project ArAGATS on the Tsaghkahovit Plain, Republic of Armenia. Archäol. Mitt. Iran Turan.

[59] Rageot M., D’Anna M.B., Huseynov M., Jalilov B., Decaix A., Berthon R., Zeng Q., Perez J., Guliyev F., Drieu L. (2026). Biomolecular analyses reveal grape-based beverages, dairy processing, and pottery function in Kura-Araxes culinary practices. Proc. Natl. Acad. Sci. USA.

[60] Decaix A., Berthon R., Mohaseb F.A., Tengberg M., Meyer J.-W., Vila E., Mashkour M., Casanova M., Vallet R. (2019). The Iranian Plateau during the Bronze Age (MOM Éditions).

[61] Davoudi H., Berthon R., Mohaseb A.F., Seno S.S., Abedi A., Mashkour M., Çakirlar C., Chahoud J., Berthon R., Pilaar Birch S. (2018). Archaeozoology of the Near East 12: Proceedings of the Groningen 2015 ASWA Conference.

[62] Hammer E. (2014). Highland fortress-polities and their settlement systems in the southern Caucasus. Antiquity.

[63] Nugent S.E., Selin Nugent C.E. (2020). Pastoralism and emergent complex settlement in the Middle Bronze Age, Azerbaijan: isotopic analyses of mobility strategies in transformation. Am. J. Phys. Anthropol..

[64] Lau H., Proctor L., Gopnik H., Bakhshaliyev V. (2020). Agropastoralism in middle bronze through early iron age Naxçıvan: Zooarchaeological and paleoethnobotanical data from Qızqala. J. Archaeolog. Sci.: Rep..

[65] Badalyan R., Smith A., Avetisyan P., Smith A.T., Rubinson K.S. (2003). Archaeology in the Borderlands: Investigations in Caucasia and Beyond.

[66] Avetisyan P. (2017). On the issues of chronology and periodisation of the Armenian Middle Bronze Age archaeological cultures. AJNES.

[67] Smith A.T. (2020).

[68] Smith A.T. (2006). Prometheus unbound: Southern Caucasia in prehistory. J. World Prehist..

[69] Sagona A. (2017).

[70] Birkett-Rees J. (2012). Power and presence: Landscape and tenure in Middle Bronze Age central Transcaucasia. Anc. Near East. Stud..

[71] Warinner C., Hendy J., Speller C., Cappellini E., Fischer R., Trachsel C., Arneborg J., Lynnerup N., Craig O.E., Swallow D.M. (2014). Direct evidence of milk consumption from ancient human dental calculus. Sci. Rep..

[72] Lindsay I., Greene A. (2013). Sovereignty, mobility, and political cartographies in Late Bronze Age southern Caucasia. J. Anthropol. Archaeol..

[73] Avetisyan P. (2014). Scientific report for the degree of Doctor of Historical Sciences (Yerevan).

[74] Göcke B., Işik K. (2014). Horses and Horse-Breeding in Urartian Civilisation. Ancient W. East.

[75] Mirzoyan L., Manaseryan N. (2017).

[76] Németh S., Kriegshäuser G., Hovhannesyan K., Hayrapetyan H., Oberkanins C., Sarkisian T. (2022). Very low frequency of the lactase persistence allele LCT-13910T in the Armenian population. Ann. Hum. Biol..

[77] Mallick S., Li H., Lipson M., Mathieson I., Gymrek M., Racimo F., Zhao M., Chennagiri N., Nordenfelt S., Tandon A. (2016). The Simons Genome Diversity Project: 300 genomes from 142 diverse populations. Nature.

[78] Wilkin S., Hagan R., Hebestreit S., Bleasdale M., Nayak A., Tang L., Billings T.N., Boivin N., Korzow Richter K. (2020). SP3 (single-pot, solid-phase, sample-preparation) protein extraction for dental calculus v1. protocols.io.

[79] Purcell S., Neale B., Todd-Brown K., Thomas L., Ferreira M.A.R., Bender D., Maller J., Sklar P., de Bakker P.I.W., Daly M.J., Sham P.C. (2007). PLINK: a tool set for whole-genome association and population-based linkage analyses. Am. J. Hum. Genet..

[80] McKenna A., Hanna M., Banks E., Sivachenko A., Cibulskis K., Kernytsky A., Garimella K., Altshuler D., Gabriel S., Daly M., DePristo M.A. (2010). The Genome Analysis Toolkit: a MapReduce framework for analyzing next-generation DNA sequencing data. Genome Res..

[81] Li H. (2013). Aligning sequence reads, clone sequences and assembly contigs with BWA-MEM. arXiv.

[82] Martin M. (2011). Cutadapt removes adapter sequences from high-throughput sequencing reads. EMBnet J..

[83] Schubert M., Lindgreen S., Orlando L. (2016). AdapterRemoval v2: rapid adapter trimming, identification, and read merging. BMC Res. Notes.

[84] Danecek P., Bonfield J.K., Liddle J., Marshall J., Ohan V., Pollard M.O., Whitwham A., Keane T., McCarthy S.A., Davies R.M., Li H. (2021). Twelve years of SAMtools and BCFtools. GigaScience.

[85] Rubinacci S., Hofmeister R.J., Sousa da Mota B., Delaneau O. (2023). Imputation of low-coverage sequencing data from 150,119 UK Biobank genomes. Nat. Genet..

[86] Hughes C.S., Moggridge S., Müller T., Sorensen P.H., Morin G.B., Krijgsveld J. (2019). Single-pot, solid-phase-enhanced sample preparation for proteomics experiments. Nat. Protoc..

[87] Hughes C.S., Foehr S., Garfield D.A., Furlong E.E., Steinmetz L.M., Krijgsveld J. (2014). Ultrasensitive proteome analysis using paramagnetic bead technology. Mol. Syst. Biol..

[88] Cleland T.P. (2018). Human bone paleoproteomics utilizing the single-pot, solid-phase-enhanced sample preparation method to maximize detected proteins and reduce humics. J. Proteome Res..

[89] Hagan R. (2018). Mpi-Shh-Mascot Report Generator (MS-MARGE). https://bitbucket.org/rwhagan/ms-marge/src/master/.

[90] Ventresca Miller A.R., Wilkin S., Bayarsaikhan J., Ramsøe A., Clark J., Byambadorj B., Vanderwarf S., Vanwezer N., Haruda A., Fernandes R. (2023). Permafrost preservation reveals proteomic evidence for yak milk consumption in the 13th century. Commun. Biol..

[91] Haber M., Mezzavilla M., Xue Y., Comas D., Gasparini P., Zalloua P., Tyler-Smith C. (2016). Genetic evidence for an origin of the Armenians from Bronze Age mixing of multiple populations. Eur. J. Hum. Genet..

[92] Antonio M.L., Weiß C.L., Gao Z., Sawyer S., Oberreiter V., Moots H.M., Spence J.P., Cheronet O., Zagorc B., Praxmarer E. (2024). Stable population structure in Europe since the Iron Age, despite high mobility. eLife.

[93] Damgaard P.d.B., Marchi N., Rasmussen S., Peyrot M., Renaud G., Korneliussen T., Moreno-Mayar J.V., Pedersen M.W., Goldberg A., Usmanova E. (2018). 137 ancient human genomes from across the Eurasian steppes. Nature.

[94] Wang C.-C., Reinhold S., Kalmykov A., Wissgott A., Brandt G., Jeong C., Cheronet O., Ferry M., Harney E., Keating D. (2019). Ancient human genome-wide data from a 3000-year interval in the Caucasus corresponds with eco-geographic regions. Nat. Commun..

[95] Bobokhyan A., Iraeta-Orbegozo M., McColl H., Mkrtchyan R., Simonyan H., Ramos-Madrigal J., Andrades-Valtueña A., Hnila P., Gilibert A., Margaryan A. (2024). Burial of two closely related infants under a “dragon stone” from prehistoric Armenia. J. Archaeolog. Sci. Rep..

[96] Jónsson H., Ginolhac A., Schubert M., Johnson P.L.F., Orlando L. (2013). mapDamage2.0: fast approximate Bayesian estimates of ancient DNA damage parameters. Bioinformatics.

[97] Auton A., Brooks L.D., Durbin R.M., Garrison E.P., Kang H.M., Korbel J.O., Marchini J.L., McCarthy S., McVean G.A., Abecasis G.R., 1000 Genomes Project Consortium (2015). A global reference for human genetic variation. Nature.

