# Supplementary material for: Palaeoproteomic and genetic insights into millennial-scale dairy consumption in Armenia
Source: iScience. 2026 Oct 9;29(11):117829. doi: 10.1016/j.isci.2026.117829 (PMC13677201; doi:10.1016/j.isci.2026.117829)
Supplement: Supplementary file 1 [file mmc1.pdf]

iScience, Volume *29*

Supplemental information

Palaeoproteomic and genetic insights into millennial-scale dairy consumption in Armenia

Mariya Antonosyan, Anahit Hovhannisyan, Patrick Roberts, Lara M. Cassidy, Satenik Mkrtchyan, Hasmik Simonyan, Arsen Bobokhyan, Levon Aghikyan, Ashot Piliposyan, Ruben Badalyan, Nzhdeh Yeranyan, Artak Gnuni, Hayk Avetisyan, Mikayel Badalyan, Armine Gabrielyan, Narineh Sarkisian, Avetis Grigoryan, Hakob Simonyan, Meri Safaryan, Benik Vardanyan, Kristine Martirosyan-Olshansky, Iseult Jackson, Emily Breslin, Andranik Gyonjyan, Zaruhi Khachatryan, Jana Ilgner, Levon Yepiskoposyan, Andrea Manica, Daniel G. Bradley, Nicole Boivin, and Shevan Wilkin

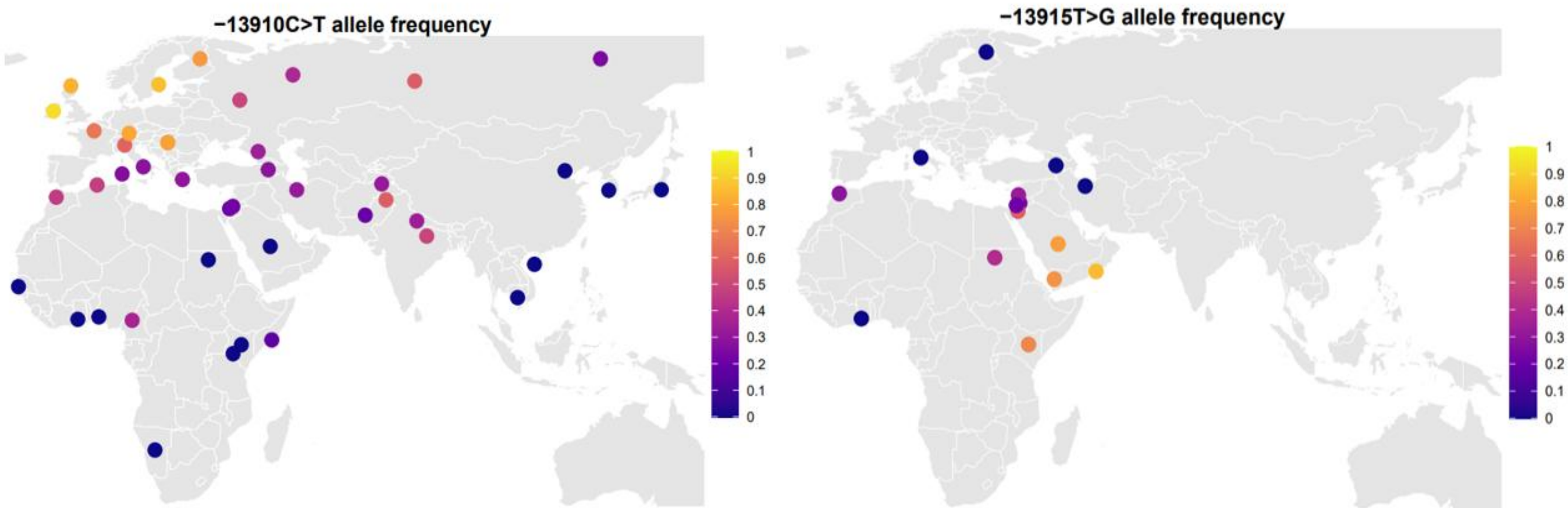

Figure S1: Map showing the frequency of LP variants in modern populations. The 13910C>T variant, common in European populations, occurs at a modest frequency in Armenians, whereas the Near Eastern 13915T>G variant is absent[13-17]

References

1. Safaryan, M. (2021). Medieval burials on the territory of the Memorial Complex Armenian Kings in Aghtsk. In: Proceedings of the History Museum of Armenia, 8. Materials of the Republican Conference dedicated to the 30th anniversary of the RA Independence, pp. 98–106.
2. Bobokhyan, A. and Kunze, R. (2021). Ushkiani-Project: preliminary archaeological investigations in the Lake Sevan region/Armenia. Praehistorische Zeitschrift 96, 500–510.
3. Aghikyan, L. and Badalyan, H. (2025). Newly discovered Early Bronze Age tomb in Dasht. In: Herald of the Social Sciences, 1, pp. 245–261.
4. Grigoryan, A., Ghulyan, A., and Miridjanyan, D. (2023). The monastery that heals from diseases: Archaeological studies in the restoring Makravank. In: Historical and Cultural Heritage of the Armenian Highland, pp. 62–63.
5. Olshansky, K. (2018). Obsidian Economy in the Armenian Highlands During the Late Neolithic: A View from Masis Blur (University of California, Los Angeles).
6. Grigoryan, A., Ghulyan, A., and Simonyan, H. (2023). Hovhan Mayravanetsi and Mayravank: Archaeological researches in the traces of medieval sources. In: Historical and Cultural Heritage of the Armenian Highland, pp. 64–65.
7. Piliposyan, A. (2014). Excavations at Metsamor. In: International Conference Problems of Early Metal Age Archaeology of Caucasus and Anatolia, Tbilisi, pp. 42–52.
8. Jakubiak, K. and Bigoraj, K. (2020). Metsamor: The Early Iron Age/Urartian settlement in the Aras Valley, Armenia. Antiquity 94, e33.
9. Simonyan, H. (2010). Nerkin Naver, Grave N 4, Hushardzan (Monument). In: Annual, 6, pp. 7–20.

10. Yeranyan, N., Zarikyan, N., and Simonyan, H. (2023). Investigation of the date and functions of Artsakh's anthropomorphic stelae in the light of the Nor Karmiravan excavations. Lraber Hasarakakan Gitutyunneri 3, 271–296.
11. Avetisyan, H., Herles, M., Wallner, M., and Zilonkowsky, J. (2014). Weitere Forschungen an der Fundstätte Oshakan, Armenien. In: Mitteilungen der Deutschen Orient-Gesellschaft zu Berlin, 146, pp. 87–138.
12. Kunze, R., Bobokhyan, A., Pernicka, E., and Meliksetian, K. (2013). Projekt Ushkiani. Untersuchungen der Kulturlandschaft um das prähistorische Goldrevier von Sotk. In: Meller, H. and Avetesyan, P. (eds), Archäologie in Armenien II – Berichte zu den Kooperationen 2011 und 2012 sowie ausgewählte Einzelstudien, Veröffentlichungen des Landesamtes für Denkmalpflege und Archäologie Sachsen-Anhalt – Landesmuseum für Vorgeschichte.
13. Bersaglieri, T., Walsh, P.T., Kashani, B.G., Dominy, N., Coward, A., Santacruz, J.D., Marks, J., and Richter, J. (2004). Genetic signatures of strong recent positive selection at the lactase gene. American Journal of Human Genetics 74, 1111–1120. 10.1086/421051.
14. Enattah, N.S., Jensen, T.G., Nielsen, M., Lewinski, R., Kuokkanen, M., Rasinperä, H., El-Shanti, H., Seo, J.K., Alifrangis, M., Khalil, I.F., et al. (2008). Independent introduction of two lactase-persistence alleles into human populations reflects different history of adaptation to milk culture. American Journal of Human Genetics 82, 57–72. 10.1016/j.ajhg.2007.09.012.
15. Mulcare, C.A., Weale, M.E., Jones, A.L., Connell, B., Whittaker, D., Bradbury, M., and Goldstein, D.B. (2004). The T allele of a single-nucleotide polymorphism 13.9 kb upstream of the lactase gene (LCT) (C−13.9 kbT) does not predict or cause the lactase-persistence phenotype in Africans. American Journal of Human Genetics 74, 1102–1110. 10.1086/420603.
16. Tishkoff, S.A., Reed, F.A., Ranciaro, A., Voight, B.F., Babbitt, C.C., Silverman, J.S., Powell, K., Mortensen, H.M., Hirbo, J.B., Osman, M., et al. (2007). Convergent adaptation of human lactase persistence in Africa and Europe. Nature Genetics 39, 31–40. 10.1038/ng1946.
17. Torniainen, S., Hedren, P., Lildbäck, E., Melin, B., Olsson, M., Savolainen, M.J., Summanen, P., Lindfors, K., Mäki, M., and Kaukinen, K. (2009). Screening of variants for lactase persistence/non-persistence in populations from South Africa and Ghana. BMC Genetics 10, 31. 10.1186/1471-2156-10-31.
